# Facile Fabrication of 100% Bio-Based and Degradable Ternary Cellulose/PHBV/PLA Composites

**DOI:** 10.3390/ma11020330

**Published:** 2018-02-24

**Authors:** Tao Qiang, Jinwu Wang, Michael P. Wolcott

**Affiliations:** 1 School of Material Science and Chemical Engineering, Xi’an Technological University, Xi’an 710021, China; 2 Composite Materials and Engineering Center, Washington State University, Pullman, WA 99163, USA; wolcott@wsu.edu; 3 Forest Products Laboratory, Forest Service, Orono, ME 04469, USA; jinwuwang@fs.fed.us

**Keywords:** cellulose, ball milling, polylactide, extrusion blending, injection molding, statistical analysis

## Abstract

Modifying bio-based degradable polymers such as polylactide (PLA) and poly(hydroxybutyrate-co-hydroxyvalerate) (PHBV) with non-degradable agents will compromise the 100% degradability of their resultant composites. This work developed a facile and solvent-free route in order to fabricate 100% bio-based and degradable ternary cellulose/PHBV/PLA composite materials. The effects of ball milling on the physicochemical properties of pulp cellulose fibers, and the ball-milled cellulose particles on the morphology and mechanical properties of PHBV/PLA blends, were investigated experimentally and statistically. The results showed that more ball-milling time resulted in a smaller particle size and lower crystallinity by way of mechanical disintegration. Filling PHBV/PLA blends with the ball-milled celluloses dramatically increased the stiffness at all of the levels of particle size and filling content, and improved their elongation at the break and fracture work at certain levels of particle size and filling content. It was also found that the high filling content of the ball-milled cellulose particles was detrimental to the mechanical properties for the resultant composite materials. The ternary cellulose/PHBV/PLA composite materials have some potential applications, such as in packaging materials and automobile inner decoration parts. Furthermore, filling content contributes more to the variations of their mechanical properties than particle size does. Statistical analysis combined with experimental tests provide a new pathway to quantitatively evaluate the effects of multiple variables on a specific property, and figure out the dominant one for the resultant composite materials.

## 1. Introduction

The effective utilization of raw natural fibers as an indispensable component in polymers for developing novel low-cost eco-friendly composites with desired physicochemical properties, such as acceptable specific strength, low density, high toughness, good thermal properties, and biodegradability, is one of the most rapidly emerging fields of research in polymer engineering and science [1]. In the field of natural fiber-reinforced composites, as far as matrix polymers are concerned, polylactide (PLA) has been regarded as the best substitute to replace “fossil carbon” with “renewable carbon” in order to manufacture green materials within the framework of environmentally-friendly processes and products [2,3,4], due to its renewability, biodegradability, biocompatibility, high strength, and good thermomechanical properties [3]. From a life cycle perspective, cassava-based PLA bottles exhibit a better environmental performance than that of their polyethylene terephthalate (PET) counterparts in terms of global warming, the reduction of dependency on fossil energy, and human toxicity [5]. Also, PLA-based composite materials show similar environmental benefits [6]. However, PLA suffers from several shortcomings, such as high cost, inherent brittleness, low thermal resistance, a slow crystallization rate, and gas permeability [7,8], which has limited its practical applications in certain scenarios.

Up to now, there are still two challenging issues for the PLA-based sustainable composites. The first issue is that it is hard to achieve a balance between stiffness and toughness for the resultant PLA composite materials [9,10], not to mention maintaining their thermal stability and other physical properties. The second issue is that most of the PLA-based composite materials stray from the 100% bioderivation and degradability of PLA, due to being modified with unsustainable and non-degradable substances [2]. In order to overcome these shortcomings and keep PLA’s merits, some completely renewable and degradable polyesters, such as polybutylene succinate (PBS) [11], poly(3-hydroxybutyrate-co-3-hydroxyvalerate) (PHBV), or poly(hydroxybutyrate) (PHB) have been used to toughen PLA. PHBV acts as nucleating agent for PLA in the resultant blends [12,13], which endows PHBV/PLA blends with proper miscibility. The miscibility between PHBV and PLA strongly depends on their individual molecular weights, their ratio in the blends, and the blending temperature [13]. There are still some disadvantages for the PHBV/PLA blends, such as its relatively high cost and unstable processability [14], although its toughness may be tunable, and perhaps even greatly improved. PHBV composite materials face the same issues as PLA [15]. In this case, natural fibers were filled into PHBV/PLA blends to produce 100% bio-based and degradable composites with the promise of extending or improving the desired properties of the resultant composite materials on the one hand, and reducing its product cost on the other hand.

Cellulose is the most widely available biopolymer, and it is also completely renewable. It is a water-insoluble and mechanically tough biomaterial that is found in nature, and plays an essential role in maintaining the structure of plant cell walls and providing the key internal load resistance to facilitate uninterrupted transport mechanisms within a plant organism. Cellulosic fibers at the micro or nanoscale are attractive replacements for man-made fibers in environmentally friendly products, due to its abundance, renewability, and degradability [16,17,18]. The applications of cellulose reinforcements in environmentally benign polymers have exhibited considerable promise for manufacturing fully bioderived polymeric composites with multiple desired functions or properties [19,20,21,22]. Previous research findings showed that cellulose microfibrils can accelerate the crystallization process of the PLA matrix [23]. Shish kebabs and transcrystallinity can form simultaneously in ramie fiber-reinforced PLA composites via continuous shear flow, which enhanced the tensile strength, tensile modulus, storage modulus, and impact toughness of the resultant composites [24]. Cellulose was grafted on PHB or PHBV in order to improve the interfacial bonding and compatibility [25]. Functionalized cellulose nanocrystals increase the thermal stability, tensile strength, Young’s modulus, elongation at break, melt-processing window, modulated crystallinity, and hydrophilic properties of the resultant PHBV nanocomposites [26]. Microcrystalline cellulose has been used to modify PLA composites through hot pressing [27] or the solution casting method [28]. However, cellulosic fibers usually experience chemical or enzymatic treatments before they are used to fabricate PLA-based composite materials [27], which will bring great complexity to the fabrication process. The solution casting method suffers from consuming a large amount of solvents and fabricating bulk polymer composites, although this approach usually well endows particles dispersion in the resultant films [4,29].

To improve the dispersion uniformity of short celluloses in molten polymer matrices and enhance the potential physicochemical properties of the resultant composites, mechanochemical activation and chemical pretreatment are usually used to break down the coiling aggregation of raw cellulose fibers, before they are filled into a polymer matrix. Chemical methods have some disadvantages for accessing such cellulose fillers for commercial production, which include batch operation, limited productivity, and complicated chemical treatments with liquid waste disposal problems [30,31]. Mechanochemical activation can break up their intra and intermolecular hydrogen bonds, and expose more reactive hydroxyl groups, which can establish new interactions with other components [32]. They facilitate the efficient disintegration of cellulose fibers by prefibrillation and fiber size reduction. Now, most of mechanochemical activation for celluloses are carried out under wet conditions [32,33]. However, the high energy use and the presence of water during wet milling lead to the increase in overall energy consumption during cellulose particle production, and pose challenges during storage and handling [34]. The development of some dry and solvent-free techniques to reduce cellulose size to a micro or nano level is an urgent demand, so that the filled composites can be easy fabricated via extrusion and injection molding processing [35]. Some physical methods, such as pan milling [32,33], ball milling [36,37], or shear and cooling milling [38], have been used to tune the aggregation state of celluloses by way of mechanical disintegration. Among all of the milling pretreatment methods, ball milling is a powerful and low-cost process that can easily introduce some changes, such as morphologies, structures, and properties, into cellulose bundles under dry conditions [35,39].

In our previous work, this facile pretreatment was used to manufacture the binary cellulose/PLA composites. The ball-milled cellulose increases the tensile modulus of the resultant composites [35]. The goal of this research is to develop 100% bio-based and degradable ternary cellulose/PHBV/PLA composite materials via such facile fabrication. Pulp cellulose fibers were ball milled in order to access the filler with appropriate physicochemical properties in a solvent-free way. Then, the effects of the ball-milled cellulose particles on the morphology and mechanical properties of the resultant composite materials were investigated experimentally and statistically.

## 2. Materials and Methods 

### 2.1. Raw Materials

Pulp cellulose fibers (southern pine, kraft bleached) were provided by Weyerhaeuser Company, Seattle, WA, USA. They were stored in a walk-in conditioning room (20 °C, 65% relative humidity) before ball milling, with a moisture content of 8.75 ± 0.22%. Ingeo PLA pellets (3052D, Tm: 145–160 °C, density: 1.24 g/cm^3^) were supplied by NatureWorks LLC, Minnetonka, MN, USA. PHBV powder, [Enmat Y1000, Tm: 165–175 °C, density: 1.24 g/cm^3^, with 8 mol % 3-hydroxyvalerate (3HV) content] was purchased from Tian’An Biologic Materials Co., Ltd., Ningbo, China.

### 2.2. Fabrication of Composites

**Figure 1:**
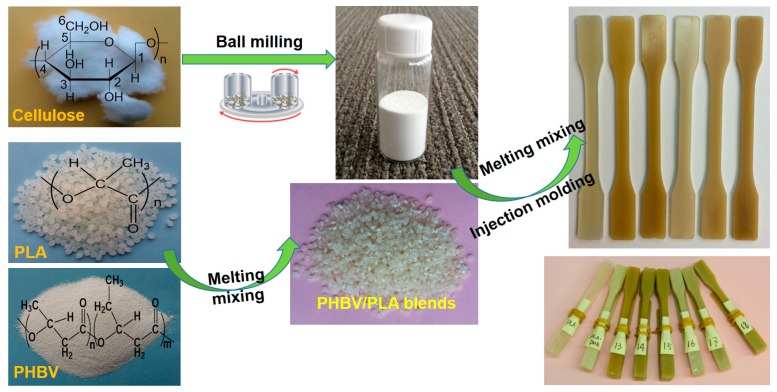
Schematic of fabricating the ternary cellulose-modified poly(hydroxybutyrate-co-hydroxyvalerate) (PHBV)/polylactide (PLA) composites.

Mechanical properties of the ternary composite materials were tuned according to the complementary physicochemical advantages of raw materials. There are three steps to fabricating the bioderived ternary composite materials, i.e., ball milling the cellulose fibers to access the filler with appropriate physicochemical properties, PHBV/PLA blending with extrusion followed by injection molding, and compound blending of cellulose/PHBV/PLA, as illustrated in Figure 1.

Cellulose fibers were treated with a planetary ball mill (Across International, PQ-N04, Livingston, NJ, USA) that runs at 600 rpm for different times under ambient conditions. Two 100 mL stainless steel jars were installed. Each of them was loaded with 3.00 g of cellulose fibers and 159.20 g of stainless steel balls (100 pieces of *Φ* 6 mm and 16 pieces of *Φ* 10 mm, respectively). During our preliminary experiments, the minimum ball-milling time to destruct raw pulp cellulose fibers was 8 min. So, we chose 10 min as the minimum ball-milling time to access cellulose fillers. After that, the ball-milling time was increased from 20 min to 90 min at a 10 min spacing to explore its effects on the aggregation state of pulp cellulose fibers.

Prior to extrusion, the ball-milled cellulose particles, PLA pellets, and PHBV powder were dried in a convection oven at 80 °C for 24 h in order to minimize the moisture content. Here, the blends of PHBV/PLA at the 25:75 weight ratio were made to get a higher miscibility between PHBV and PLA, referring to Arrieta’s results [13], with a twin-screw extruder (HAAKE MiniLab II, Thermo Scientific, Waltham, MA, USA) running at 75 rpm and 180 °C for 5 min. Hereinafter, the PHBV/PLA blends will be the control.

After cooling with water, the PHBV/PLA extrudes were chopped into pellets with a strand pelletizer (BT25, Bay Plastics Machinery, Bay City, MI, USA), and dried in the oven at 80 °C overnight. The extruded PHBV/PLA blends were injection-molded to get the control composites (HAAKE MiniJet, Thermo Electron, Newington, CT, USA). Respectively, the cylinder and mold temperature were 190 °C and 70 °C, and the injection and holding pressure were 600 and 400 bar, respectively. The injection and holding times were 10 s and 30 s, respectively. Then, a series of the PHBV/PLA blended pellets and ball-milled cellulose particles (4.8 wt %, 13.0 wt %, and 20.0 wt % of cellulose, respectively) were extrusion-blended, chopped, dried, and injection-molded at the same processing conditions as that of the control (i.e., the PHBV/PLA blends). The components of the ternary cellulose/PHBV/PLA composite materials were shown in Table 1.

### 2.3. Characterization

A laser diffraction analyzer (Mastersizer 3000, Malvern Instruments Ltd., Malvern, UK) was used to measure the particle size and size distribution of the ball-milled celluloses, with distilled water used as the dispersant. An X-ray diffractometer (MiniFlex 600, Rigaku, Japan) was used to investigate their crystallinity, with a scanning speed of 2.0000 °/min, according to Segal’s empirical method [40]. The crystallinity of cellulose was calculated by subtracting the amorphous contribution approximately at 2*θ* = 18°, i.e., [(*I*_22.6_ − *I*_18_)/*I*_22.6_]. A Fourier transform infrared spectrometer (FTIR; NEXUS, Thermo-Nicolet, Madison, WI, USA) was used to record the changes in the characteristic absorption bands, running from 4000 cm^−1^ to 400 cm^−1^ at 8 cm^−1^ spectral resolution for 32 scans in the absorbance mode.

A tensile test with five valid replicates was carried out under ambient conditions (Instron 4466, Norwood, MA, USA) at a crosshead rate of 5.08 mm/min, according to ASTM D638 (Type I). The initial strain was measured with a two-inch (50.8 mm) extensometer. The fracture work of each tensile bar was calculated according to the area integration under their individual stress–strain curve. An environmental scanning electron microscope (ESEM; Quanta 200, FEI, Hillsboro, OR, USA) was used to investigate the cellulose morphology and tensile fractured surfaces of the resultant composites, after coating a conducting layer with a sputtering coater (Cressington, 208 HR, Redding, CA, USA). The thickness of platinum layer was about 10 nm when the spraying time was set at 5 min.

### 2.4. Statistical Analysis

An analysis of variation (ANOVA) and correlation analysis were conducted to verify the contributions of celluloses to the mechanical properties of the resultant composite materials (QI Macros, 180-day trial version, KnowWare International Inc., Denver, CO, USA and SPSS, version 17.0, IBM, Armonk, NJ, USA, respectively). 

## 3. Results and Discussion

### 3.1. Ball Milling on the Cellulose Fibers

**Figure 2:**
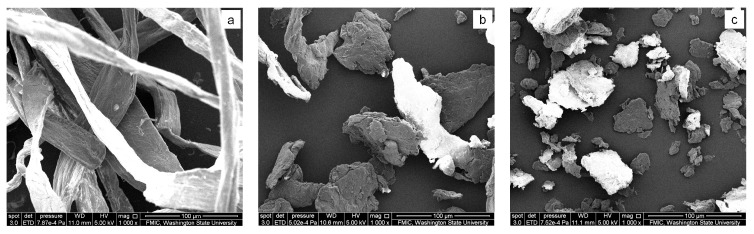

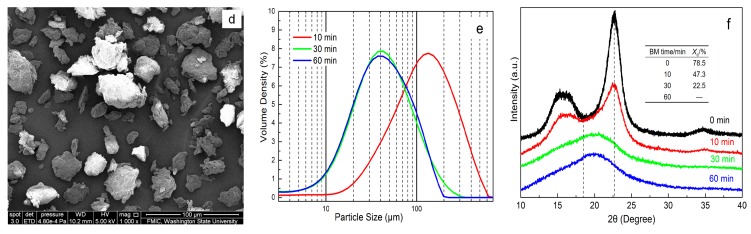
Effects of ball milling on the cellulose fibers. Panel (**a**) shows the raw cellulose fibers under SEM. Panels (**b**–**d**) illustrate the cellulose particles with ball-milling times of 10 min, 30 min, and 60 min, respectively. Magnification of the SEM image is 1000, and the scale bar is 100 μm. Panels (**e**, **f**) show the particle size distribution and wide-angle X-ray diffraction curves of the ball-milled cellulose particles, respectively.

There are some fibrils on the surfaces of the single raw pulp cellulose fibers, as shown in Figure 2a. These fibers are entangled with each other, due to their high aspect ratio, so they cannot be used to fill polymer directly via extrusion or injection molding processing. The ball-milling process destructed these loose, interentangled pulp cellulose fibers, and turned them into irregular particles, due to intense mechanical stress [36]. Ball milling the raw cellulose fibers for 10 min produced cellulose particles with an average size of 120.0 μm (Figure 2b), which makes it possible for them to be used as a filler or modifier for polymeric composites via the traditional melting process, such as extrusion and injection molding. The longer ball-milling time contributed to not only a smaller particle size and narrower particle size distribution, but also more regular particle shapes (Figure 2b–d). However, the size reduction effect became no longer obvious when the ball-milling time was more than 30 min. For example, an increase in the ball-milling time from 30 min to 60 min only lead to a 0.8-μm decrease on the average particle size (from 39.7 μm to 38.9 μm, see Figure 2c–e).

Mechanical ball milling also influenced the crystallinity of the pulp cellulose fibers. The crystallinity of the raw cellulose fibers was 78.5%, while the counterpart with 10 min of ball milling reduced to 47.3% (Figure 2f), according to the Segal method [40]. Cellulose fibers became amorphous when the ball-milling time reached 30 min. These results are consistent with some previous findings [41]. The cellulose particles with different aggregation states can be used to tune the desired morphology and physical properties for some expected composites [42].

**Figure 3:**
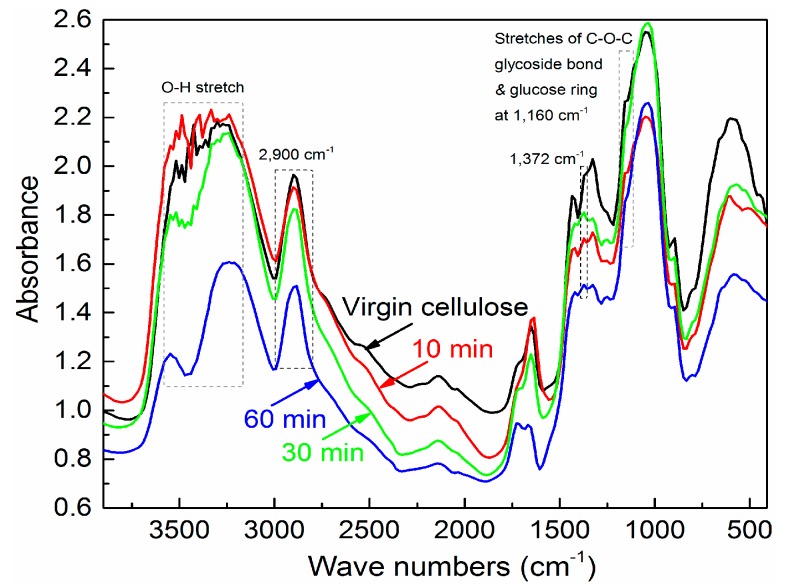
Fourier transform infrared (FTIR) spectra of the virgin and ball-milled pulp cellulose fibers.

The effects of ball milling on cellulose fibers can also be observed via FTIR analysis [43]. As shown in Figure 3, both the shape changes of the FTIR spectra over two ranges (3700–2800 cm^−1^ and 1500–600 cm^−1^, respectively) and a slight position shift of band maxima were observed for the ball-milled cellulose particles, compared with that of the virgin pulp cellulose fibers. Here, the most conspicuous changes were in the broad range from 3200 cm^−1^ to 3650 cm^−1^, and at the wavenumbers of 1160 cm^−1^, which are assigned to the characteristic stretching of intra and intermolecular hydrogen bonds, and the deformation vibrations of the C–O–C bond and the glucose ring, respectively. They were sensitive to the ball-milling process [43]. The relative band heights of the band at 1160 cm^−1^ decreased, while the OH-stretching vibration region became broader as the ball-milling time increased.

The changes in the FTIR spectra for the cellulose fibers due to ball milling can be associated with changes in the degree of crystallinity, depending on the milling time. Several attempts have been made to quantify its degree of crystallinity, according to the intensities of certain bands in the infrared spectra, such as the ratio of band heights at 1372 cm^−1^ and 2900 cm^−1^ [44]. The crystallinity of the virgin cellulose fibers was 79.2%, and those of the ball-milled cellulose particles were 46.9% (10 min), 21.1% (30 min), and 14.2% (60 min), respectively, according to the *A*_1372_/*A*_2900_ method [44]. The calculated crystallinities of raw cellulose fibers and ball-milled cellulose particles for 10 min and 30 min are close to the values from our previous X-ray diffraction. In fact, the 30-min ball-milled cellulose particles became amorphous, even though they illustrated a relative crystallinity of 21.1%, according to the *A*_1372_/*A*_2900_ method.

Ball milling raw cellulose fibers at room temperature, without any other chemical treatments, is a difficult method for obtaining nanometer-sized particles [45], though it has strong influences on the aggregation state. Ball milling is a mechanical disintegration process for obtaining micrometer cellulose particles, because there is no new characteristic absorption band appearing in their FTIR spectra, which only brings a size-reduction effect to the cellulose fibers [38]. Herein, three different levels of ball-milling time (i.e., 10 min, 30 min, and 60 min, respectively) were selected to access different types of cellulose fillers for the PHBV/PLA composite materials: big and crystalline particles, middle and amorphous particles, and small and amorphous particles.

### 3.2. Mechanical Properties of the Composites

**Figure 4:**
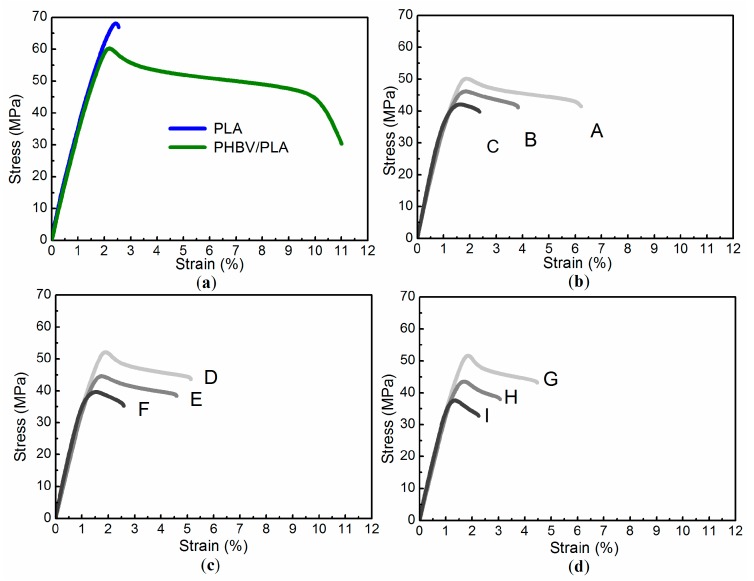
Typical stress–strain curves of the cellulose/PHBV/PLA ternary composites, with that of PLA and PHBV/PLA as reference. Panel (**a**) describes stress–strain curves of PLA and PHBV/PLA. Panel (**b**) shows that of Sample A, B, and C, respectively. Panel (**c**) is that of Sample D, E, and F, respectively. Panel (**d**) describe that of Sample G, H, and I, respectively.

Typical stress–strain curves for PLA, PHBV/PLA blends, and the ternary composites were randomly selected from their five repeated tensile bars, as shown in Figure 4. The inclusion of PHBV into PLA significantly improved the ductility of PLA, with the compromise of tensile strength, since PHBV has a plasticization effect on PLA at a high PHBV/PLA mass ratio (25:75). Transesterification reactions might take place between PLA and PHBV chains in the melt state, which make the blends highly miscible. The stress–strain curves show that whatever the particle size, low cellulose filling (i.e., 4.8 wt %) performs the best in mechanical property enhancements of the resultant ternary composites, while a high cellulose content badly deteriorates both ductility and tensile strength.

**Figure 5:**
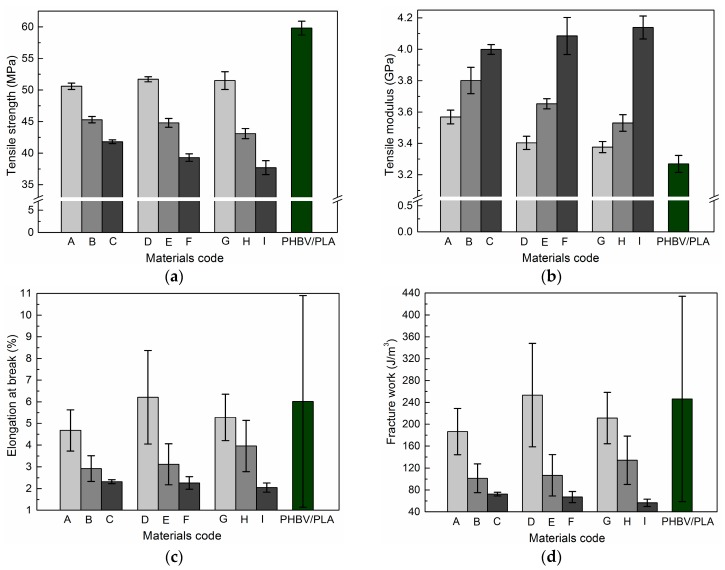
Tensile strength, tensile modulus, elongation at break, and fracture work of the ternary cellulose/PHBV/PLA composite materials, compared with that of the PHBV/PLA blends as reference. Panel (**a**–**d**) show tensile strength, tensile modulus, elongation at break, and fracture work, respectively.

The average tensile yield strength, Young’s modulus, elongation at break, fracture work, and their individual standard deviations are shown in Figure 5. The stiffness of the cellulose/PHBV/PLA composites dramatically increased due to the cellulose’s reinforcement of the PHBV/PLA blends (see the increase of Young’s modulus in Figure 5). Besides that, the elongation at break and fracture work of the cellulose-modified PHBV/PLA ternary composites were slightly improved at certain levels of particle size and filling content, compared with that of the PHBV/PLA blends, which is ascribed to the chain entanglements and hydrogen bonding interactions between the miscible matrix (i.e., PHBV/PLA) and the filler (i.e., ball-milled celluloses) [26].

The error bars in Figure 5 represent their individual standard deviations, which reflect the dispersion degree of the measure mechanical properties. The higher the standard deviation, the more the measured values deviated from their average. From the point view of product quality, raw materials with higher standard deviations in mechanical properties usually lead to unreliable and inconsistent quality. The standard deviations of the above-mentioned mechanical parameters for the ternary composite materials were greatly reduced, compared with that of the control (i.e., the PHBV/PLA blends). It means that filling the ball-milled cellulose particles into the PHBV/PLA blends leads to smaller standard deviations of mechanical properties, compared with that of the control. Thus, products made out of the ternary cellulose/PHBV/PLA composites will have more reliable and consistent quality than those made out of the PHBV/PLA blends.

Low cellulose filling kept almost the same plastic deformation resistance (yield strength) and ductility (elongation at break and fracture work) for the resultant ternary composites as that of the PHBV/PLA blends, when the particle size decreased from 120.0 μm to 38.9 μm. The reason is that the ternary composite materials with a low cellulose filling introduce less interfacial defects, such as debonding and voids. So, they exhibit the positive effects on the mechanical property improvements, which will be investigated hereinafter via fractured surface analysis.

Clearly, the ball-milled pulp cellulose particles can be used as filler to produce 100% bio-based and degradable polymer composites with tunable mechanical properties within a certain range for some potential application scenarios. Furthermore, it seems that the filling content contributed more to mechanical property enhancements than particle size did, which will be investigated hereinafter with statistical analysis methods. The mechanical properties illustrated by the ternary cellulose/PHBV/PLA composites hint at some potential applications, such as packing materials and automobile inner decoration parts.

### 3.3. Tensile Fractured Surfaces

**Figure 6:**
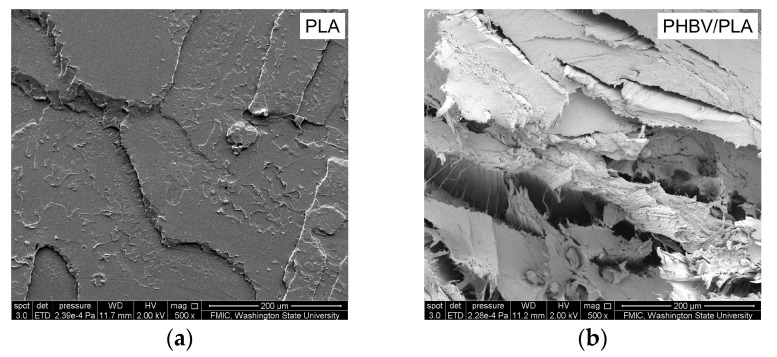
SEM images of the tensile fractured surfaces from PLA (**a**) and PHBV/PLA blends (**b**). The scale bar represents 200 μm, and magnification is 500.

The tensile fractured surfaces of the PHBV/PLA blends were different from that of PLA, in that virgin PLA demonstrates inherent stiff and brittle fractured surfaces (Figure 6). In the blends, PLA and PHBV show high miscibility at a proper weight ratio (i.e., 25:75), especially in the melt state, due to the transesterification reactions that take place between the PLA and PHBV chains [13].

**Figure 7:**
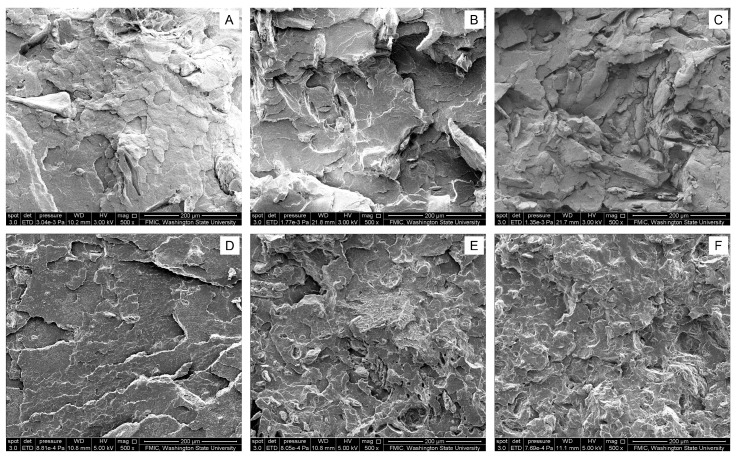

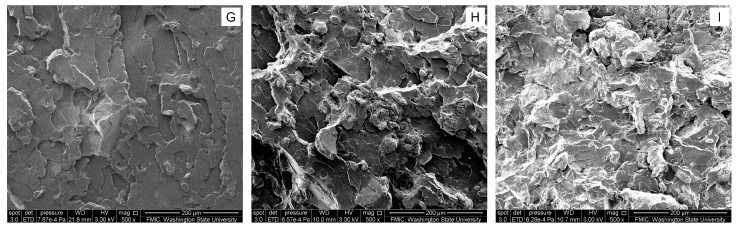
SEM images of the tensile fractured surfaces from the cellulose/PHBV/PLA composite materials. Magnification is 500, and the scale bar represents 200 μm.

The global tensile fractured surfaces of the ternary cellulose/PHBV/PLA composite materials become rougher and more irregular as the filling content increases and the particle size decreases, as illustrated in Figure 7 from the upper left to the lower right. There are some cracks distributed uniformly on the fractured surfaces, showing a hybrid pattern combining with delamination and pull-out. The ternary composites with low cellulose filling (i.e., 4.8 wt %) illustrated the delamination pattern, which is attributed to the proper miscibility of PHBV/PLA matrix [13]. Besides that, more and more microcracks and microvoids were noticed on their fractured surfaces for the ternary composites with smaller cellulose particles and a higher filling content. The interfaces between the thermoplastic matrix (i.e., the PHBV/PLA blends) and fillers (i.e., the ball-milled cellulose particles) became more uneven and intricate. Their interfacial adhesions were greatly improved compared to that in PLA/Cordenka or PLA/flax composites [9], due to the existence of PHBV.

As far as local fractured surfaces are concerned, the zone of PHBV/PLA blends demonstrated the delamination pattern, while that of cellulose particles mainly showed a pull-out pattern. Pulp cellulose fibers are hydrophilic and polysaccharide in nature. Ball-milling raw cellulose fibers at room temperature, without adding any other chemicals, is a mechanical disintegration process that only reduces their size [38]. Thus, microphase separation will take place between the hydrophilic celluloses and the hydrophobic PHBV/PLA blends, which introduces some microcracks into their tensile fractured surfaces. The bonding between the ball-milled cellulose particles and the PHBV/PLA matrix might be greatly enhanced, if some chemical methods were taken to pretreat the cellulose fibers in order to increase its hydrophobicity.

### 3.4. Statistical Analysis

According to our tensile test results, it seemed that filling content and particle size had unequal contributions to the mechanical property improvements for their resultant composites, which will be investigated with statistical analysis methods. An analysis of variation (ANOVA) with two-factor and replication has been used to determine the influence of some variables on others [46]. The effects of filling content and particle size on the mechanical properties of the cellulose-reinforced PHBV/PLA blends via ANOVA are tabulated in Table 2. The results show that all of the computed Fisher values (*F* in Table 2) that are related to filling content are higher than the critical Fisher value (here, 5.248), while two of the *F* values related to particle size are lower than the critical Fisher value (also 5.248). The same conclusion can be drawn according to their individual *p*-values. All of the *p*-values related to filling content are smaller than the significance level (i.e., 0.01), while two of the values related to particle size are larger than 0.01. This means that the filling content and particle size did have unequal contributions to the variation of their mechanical properties for the resultant ternary composite materials.

The Pearson correlation coefficient has been used to determine the effects of some variables on the mechanical properties of composite materials [47,48]. As shown in Table 3, the correlation coefficients between the filling content and the mechanical properties were much higher (the minimum absolute value, 0.784) than those between the particle size and the mechanical properties (the maximum absolute value, 0.155). The results show that as far as correlation coefficients are concerned, the filling content contributes more to the variations in mechanical properties than particle size, which verified our preliminary conclusion from mechanical tests.

The filling content of cellulose has a significantly positive correlation to Young’s modulus, and significantly negative correlations to yield strength, elongation at break, and fracture work. It is consistent with the tensile test results that are shown in Figure 5. On the contrary, particle size is believed to be uncorrelated to the mechanical properties of the resultant composites. Statistical analysis combining with experimental results can be used to evaluate the individual contributions of multiple variables on the specified properties, and figure out the dominant variable during the materials’ development process.

## 4. Conclusions

In this research, a solvent-free and facile route was developed to fabricate 100% bio-based and degradable ternary cellulose/PHBV/PLA composites with tunable mechanical properties and improved processing stability. The results show that ball milling the loose pulp cellulose fibers under ambient conditions is a viable pretreatment to access the cellulose fillers to manufacture low-cost and renewable polymer composites with tunable mechanical properties. Filling the PHBV/PLA blends with the ball-milled celluloses dramatically increases their tensile modulus, while decreases their tensile strength at all of the levels of particle size and filling content. The low filling content of cellulose keeps almost the same plastic deformation resistance and ductility for the resultant ternary composites as that for the PHBV/PLA blends, whatever the particle size. The elongation at break and fracture work are enhanced for Sample 4, compared with that of the PHBV/PLA blends. The ternary cellulose/PHBV/PLA composites have some potential applications, such as in packaging materials and automobile inner decoration parts. The filling content contributes more variation to the mechanical properties for the resultant composites than particle size, according to the two-factor ANOVA. Statistical analysis combined with experimental tests could be used to quantitatively estimate the individual effects of multiple variables on the specified properties of the resultant composites and point out the dominant one, which would be a new way of optimizing the design and fabrication of polymeric composite materials in the future.

## Figures and Tables

**Table 1:** Components of the ternary cellulose/PHBV/PLA composites.

Materials Code	Content of Cellulose/PHBV/PLA/wt %	Average Particle Size of Cellulose/μm
Sample A	4.8/23.8/71.4	120.0
Sample B	13.0/21.8/65.3	
Sample C	20.0/20.0/60.0	
Sample D	4.8/23.8/71.4	39.7
Sample E	13.0/21.8/65.3	
Sample F	20.0/20.0/60.0	
Sample G	4.8/23.8/71.4	38.9
Sample H	13.0/21.8/65.3	
Sample I	20.0/20.0/60.0	

**Table 2:** Results of ANOVA.

Factor	Mechanical Property	*F*	*p*-Value
Particle size	Tensile strength *	20.15	0.000
	Tensile modulus *	11.47	0.000
	Elongation at break	1.247	0.299
	Fracture work	0.9949	0.380
Filling content	Tensile strength *	829.7	0.000
	Tensile modulus *	378.6	0.000
	Elongation at break *	37.04	0.000
	Fracture work *	47.04	0.000

Note: * means significant at the 0.01 level (two-tailed).

**Table 3:** Results of Pearson correlation coefficients.

Factor	Mechanical Property	Pearson Coefficients	Significance
Particle size	Tensile strength	0.116	0.447
	Tensile modulus	0.155	0.309
	Elongation at break	−0.143	0.349
	Fracture work	−0.111	0.468
Filling content	Tensile strength *	−0.962	0.000
	Tensile modulus *	0.904	0.000
	Elongation at break *	−0.784	0.000
	Fracture work *	−0.815	0.000

Note: * means significant at the 0.01 level (two-tailed).
